# The Dental Protective Association

**Published:** 1893-06

**Authors:** 


### THE DENTAL PROTECTIVE ASSOCIATION.

On another page is given a communication from the president of this Association, Dr. Crouse.

The work of this organization has been so positive in good that it seems unnecessary to utter a word in its behalf. Yet the large number of dentists who have failed to join it seems to make this necessary.

The many who still entertain a doubt of its value must be convinced by reading this report that the small fee charged will prove the best investment that can possibly be made.

The promises of this Association for the future indicate a wide extension of its work, so wide that but for the well-known energy of the president we might be disposed to question the possibility of success.

The past work is quite sufficient to prove a guarantee of usefulness for the future, but in order to make this more positive every dentist in this broad land should be upon its books.

